# Fly Ash Utilisation for CO_2_ Reduction in Cement Composites

**DOI:** 10.3390/ma19081490

**Published:** 2026-04-08

**Authors:** Jakub Sobala, Jakub Szczurowski, Danutė Vaičiukynienė, Ignasi Casanova, Paweł Baran, Katarzyna Zarębska

**Affiliations:** 1 Faculty of Energy and Fuels, AGH University of Krakow, 30-059 Kraków, Poland; kubaeip@agh.edu.pl; 2 Faculty of Civil Engineering and Architecture, Kaunas University of Technology, LT-44249 Kaunas, Lithuania; danute.vaiciukyniene@ktu.lt; 3 Department of Civil and Environmental Engineering, Universitat Politècnica de Catalunya, 08034 Barcelona, Spain; ignasi.casanova@upc.edu; 4 Instytut Mechaniki Górotworu Polskiej Akademii Nauk, 30-059 Kraków, Poland; pawel.baran@imgpan.pl; 5 Faculty of Environmental Engineering, Geomatics and Renewable Energy, Kielce University of Technology, 25-314 Kielce, Poland; kzarebska@mail.tu.kielce.pl

**Keywords:** CO_2_ emission reduction, fly ash, cement composites, circular economy, decarbonisation

## Abstract

**Highlights:**

- Compared two fly ash types (F1 and F2) in cement composite synthesis.
- Demonstrated effective fly ash activation using Portland cement.
- Achieved 41 MPa compressive strength with 25–50% cement reduction.
- Enhanced mesoporosity and surface area via BET and BJH analyses.
- Confirmed pozzolanic activity through XRD, FTIR, and TGA characterizations.

**Abstract:**

This study examines the utilisation of fly ash from the energy sector as a secondary raw material in cement composites, with the aim of improving sustainability while maintaining high mechanical performance. By partially replacing Portland cement with industrial by-products, the proposed approach supports resource efficiency and aligns cement composite production with circular economy principles. Three formulations were tested: a reference mix and mixes with 25% and 50% cement reduction. Compressive strength reached 41 MPa, confirming suitability for construction use. Chemical and textural properties were analysed using XRD, FTIR, TGA, and nitrogen adsorption (BET, BJH). The results showed structural modifications, including new crystalline phases and changes in porosity. XRD confirmed newly formed phases, while FTIR identified Si-O-Si and Al-O-Si bonds, indicating effective activation of fly ash. Reducing cement content increased surface area and mesoporosity, enhancing performance. The findings demonstrate that fly ash can serve as a sustainable substitute for Portland cement within a circular economy framework, supporting CO_2_ emission reduction and resource conservation while enabling the production of durable and environmentally responsible cement composites.

## 1. Introduction

As the world moves toward carbon neutrality and decarbonisation, reducing greenhouse gas emissions from key industrial sectors has become a major international priority. The European Union countries are expected to achieve net zero carbon dioxide emissions by 2050 [1]. The cement industry is responsible for approximately 7% of worldwide CO_2_ emissions [2]. This significant contribution makes it a key sector within the common efforts towards decarbonisation. This objective is supported by several recent initiatives and projects, including the cement industry [3,4]. The Cement Technology Roadmap: The low carbon transition in the cement industry was formulated by the International Energy Agency (IEA) in 2009 and subsequently updated in cooperation with the Cement Sustainable Industries Initiative (CSI) and the World Business Council for Sustainable Development (WBCSD). The fundamental premise of the project is that by 2050, the amount of carbon dioxide emitted will be reduced by 50%, and the cement sector itself will reduce its CO_2_ emissions by up to 18% [5,6]. The project was implemented in four distinct phases: energy efficiency, material change, the use of alternative fuels and carbon capture and storage technology (CCS) [7].

Given the fundamental role of cement in modern infrastructure, its production cannot be eliminated. Therefore, current strategies focus on reducing its environmental footprint through energy efficiency, alternative fuels, carbon capture technologies, and, importantly, the partial substitution of clinker with supplementary materials [8,9]. One of the most promising approaches involves the utilisation of industrial residues, particularly fly ash generated during fossil fuel combustion. Its chemical properties reduce raw material use and waste, while proper application supports environmental protection, circular economy principles, and sustainable development [10,11]. Using this material as a raw material reduces greenhouse gas emissions and lowers cement production costs. It also improves plant efficiency and decreases landfill accumulation [12,13].

Fly ash can be chemically activated using concentrated alkalis and sodium silicate to produce porous materials with applications in construction, agriculture, wastewater treatment, and the energy sector. The chemical activation process increases the reactivity of fly ash, enabling it to form stable structures that provide durability and sorption properties that are valuable in various industrial applications [14]. The chemical mechanism of the reaction varies depending on the activator used. In the context of geopolymerisation, the most prevalent activators comprise concentrated solutions of sodium and potassium hydroxides, water glass, and salt admixtures. These activators give rise to the formation of a three-layer amorphous geopolymer network. The geopolymerisation process comprises three distinct stages: nucleation, oligomerisation and polymerisation [15]. The synthesis of zeolites using fly ash is also based on the use of concentrated alkaline solutions in the case of the hydrothermal method. However, in this instance, the process is more complex, consisting of four distinct stages: dissolution of aluminosilicates, condensation, gelation and crystallisation [16,17]. In slag and composite cement systems, sodium-based activators such as NaOH, Na_2_CO_3_, and Na_2_SiO_3_ play a crucial role. Sodium silicate has been found to promote rapid setting and early strength due to its dense microstructure, while sodium carbonate has been observed to delay setting [18]. Activators accelerate hydration reactions and encourage the formation of C-S-H and C-A-S-H gel phases, which seal the microstructure. This increases the reactivity of combustion by-products, enabling a more balanced composite binder to be obtained [19]. High concentrations of alkalis, such as NaOH and KOH, serve as effective activators, but also present toxicity risks and potential environmental hazards if not managed properly. In response, attention has been shifted to alternative activators such as Portland cement and Ca(OH)_2_, which are considered safer. These safer compounds offer a viable solution to reduce the environmental impact of the synthesis process, although they can increase production costs and sometimes yield materials with slightly different properties [20,21].

The specific surface area of fly ash strongly influences its pozzolanic activity in cement mixtures. Malhotra and Mehta [22] demonstrated that the pozzolanic activity of fly ash is strongly dependent on its specific surface area, with a significant increase in reactivity observed only at higher values (0.20–1.00 m^2^/g), while lower surface areas result in limited contribution to cement hydration. Recent studies emphasise the importance of precise determination of fly ash surface area and textural parameters for evaluating its pozzolanic reactivity. Wang et al. [23] showed that specific surface area values obtained using the BET method (5–20 m^2^/g) are significantly higher than those determined by the Blaine method (0.20–0.45 m^2^/g), as BET accounts for microporosity that is not represented by Blaine measurements. In addition, image analysis was identified as a reliable alternative, providing comparable results while also offering insights into particle morphology.

Chemical activation of fly ash significantly affects the kinetics of the hydration reaction. Kuryłowicz-Cudowska et al. [24] found that a 20% replacement of cement with ash reduced the total heat of hydration after 165 h from approximately 310 J/g (reference sample, 296 K) to approximately 260 J/g, while at 326 K these values were 355 J/g and 320 J/g, respectively. At the same time, the induction time increased, and the maximum heat release rate decreased. In another study, reaction rate constants were higher for mortars with 30% fly ash than for those with 40%, indicating faster strength development at lower ash content under the same thermal conditions. These results clearly indicate that analysis of the heat of hydration and kinetics allows quantitatively capturing the differences in fly ash activation mechanisms and their influence on properties in the early stages [25].

Assessing the carbon footprint (CF) has become an essential element in manufacturing sectors, enabling companies to evaluate and manage their environmental impact across the entire product life cycle. In this context, Life Cycle Assessment (LCA) is widely used as a structured approach for quantifying environmental impacts, including CO_2_ emissions, at different stages of production. The importance of such analyses is further emphasised by current regulatory frameworks, such as the European Green Deal, the Corporate Sustainability Reporting Directive (CSRD), and the EU Taxonomy [26].

In the construction industry, LCA plays a key role in evaluating the environmental performance of materials, including systems incorporating fly ash. By identifying stages with the highest environmental burden, it enables targeted improvements and supports the development of more sustainable materials. At the same time, considering the environmental and health concerns associated with conventional alkaline activation methods, alternative approaches based on Portland cement or calcium-rich compounds are increasingly recommended. These solutions are less hazardous and more practical for large-scale applications. A relevant example is the synthesis of cement–fly ash composites, which allows effective utilisation of industrial by-products while improving the environmental profile of construction materials [27,28,29,30].

According to the literature, the use of fly ash in the production of building materials can significantly reduce the carbon footprint. Several studies have reported CO_2_ emission reductions of up to 63% compared to conventional OPC-based concrete, depending on the material composition and strength class [31]. Similarly, LCA studies of geopolymer systems indicate reductions in global warming potential (GWP) of up to 49.7%, although the results may vary depending on factors such as transport and the type of activator used [32]. Partial replacement of cement with combustion by-products at levels of 40–50% has been shown to reduce emissions by approximately 30% [33]. Furthermore, LCA studies comparing alternative construction materials, such as compressed stabilised earth blocks containing fly ash, with conventional fired ceramic bricks demonstrate reductions in CO_2_ emissions of up to ~80%, highlighting the environmental and economic advantages of such solutions [34]. These findings support the environmental potential of cement–fly ash composites developed in this study, which achieve compressive strengths of up to 41 MPa with a 25–50% reduction in cement content, contributing to more sustainable construction and circular economy principles.

The use of fly ash offers diverse economic benefits, depending on its application across various sectors. In construction, incorporating fly ash as a cement and concrete additive can reduce production costs by up to 30%, while simultaneously lowering CO_2_ emissions [35]. In contrast, the production of lightweight aggregates from fly ash involves higher energy consumption but yields high-value products that can offset initial investment costs [36]. The economic viability of fly ash utilisation depends strongly on raw material availability. In coal-reliant countries, abundant and low-cost fly ash enables cost-effective applications across various industries, supporting the wider adoption of sustainable practices. In regions undergoing an energy transition, however, decreasing availability may increase acquisition costs, requiring more efficient and targeted utilisation strategies based on local market conditions [37]. Table 1 compares fly ash from hard coal and lignite, highlighting compositional differences that influence their economic value and practical applicability in cement-based materials [38,39].

Previous studies have investigated the influence of fly ash on cement hydration, strength development, and carbonation behaviour. It has been shown that increasing fly ash content generally reduces the heat of hydration and modifies reaction kinetics, particularly at early curing stages. At the same time, fly ash contributes to long-term strength development through pozzolanic reactions and microstructural refinement. Its incorporation may also affect carbonation processes, depending on the composition of the ash and curing conditions. Furthermore, recent studies indicate that fly ash contributes to the immobilisation of heavy metals in cement-based materials, highlighting its potential in environmentally oriented applications [40,41,42,43,44].

The contemporary energy sector, largely based on fossil fuel combustion, generates significant amounts of waste, including fly ash. Derived from coal and lignite combustion, fly ash is widely used in construction and civil engineering, particularly in concrete production and soil stabilisation. Hard coal fly ash, rich in silica and alumina, is suitable as a cement additive, while lignite ash, with higher calcium content, can partially replace clinker. Despite its advantages, fly ash may contain heavy metals, requiring proper assessment before use. Its utilisation supports waste management and enables broader industrial applications [45,46,47].

The objectives of this study were to develop a methodology for utilising combustion by-products from the power sector in the production of cement–fly ash composites and to compare two fundamentally different types of fly ash derived from hard coal and lignite.

The novelty of this work lies in the systematic and integrated investigation of source-specific variations in the reactivity, phase composition, and textural properties of fly ashes. These features are directly linked to the performance and properties of the resulting cement–ash composites. In contrast to previous studies, which typically focus primarily on chemical composition and mechanical performance, this work explicitly incorporates a detailed analysis of porous texture, including specific surface area and pore size distribution (PSD), as key parameters governing material behaviour. To achieve this, a multi-technique characterisation approach was employed, including XRD, FTIR, TGA, and nitrogen adsorption analyses (BET and BJH methods), enabling detailed insights into phase transformations, chemical bonding, and the development of micro- and mesoporous structures. The experimental methodology involved three series of materials synthesised using Portland cement 42.5R as an activating agent, all of which achieved high compressive strength after stabilisation.

Particular emphasis was placed on understanding the role of cement-based activation in promoting favourable bonding, phase formation, and microstructural evolution, with direct implications for durability and mechanical resistance. Furthermore, this study provides a comparative evaluation of fly ash derived from hard coal and lignite, highlighting how differences in their composition and origin influence both the porous structure and the performance of cement-based composites.

## 2. Materials and Methods

### 2.1. Experimental Materials

**Figure 1:**
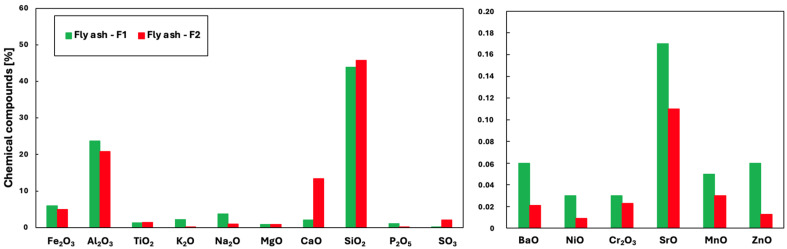
Chemical composition of fly ash.

This study used fly ash samples from different industrial energy sectors (designated as F1 and F2). These samples were combined with classic Portland cement (PC) 42.5R. The physicochemical properties of the cement are summarised in Table 2. The selection of these components was based on their complementary properties, which was intended to enhance the performance of the resulting composites. The chemical composition of the fly ash samples was determined using X-ray fluorescence spectroscopy (XRF), using an EDXRF Epsilon 3XLE spectrometer, Malvern Panalytical Ltd., Malvern, UK. The results summarised in Figure 1 indicate the concentrations of main metal oxides. Fly ash exhibited a silicon oxide (SiO_2_) content between 43% and 45%. Aluminium oxide (Al_2_O_3_) was present at concentrations of 20–25% in fly ash derived from coal. Iron oxide (Fe_2_O_3_) concentrations were below 6% in fly ash from coal and lignite combustion. In particular, fly ash from lignite combustion exhibited the highest calcium oxide (CaO) content at 13%. Fly ash samples with a CaO content greater than 10% by weight were classified as high calcium fly ash (HCFA), due to their pozzolanic properties and potential for application in cementitious composites.

The ash contains heavy metals mainly in the form of oxides. The content of potentially toxic elements such as Ni, Cr, and Sr typically remains below 0.1 wt%. Although the total concentrations of potentially toxic elements (Ni, Cr, Sr) are below 0.1 wt%, their leaching behaviour in the cementitious matrix was not assessed in this study.

The integration of these materials into composite formulations was aimed at optimising the chemical and mechanical properties of the end products, exploiting the different oxide compositions for improved reactivity and stabilization.

#### 2.1.1. Preparation of Cement Pastes

Samples were prepared using a cement mortar mixer according to PN-EN 196-1 [48] (“Methods for Testing Cement, Part 1: Determination of strength”), manufactured by GEOLAB, Warsaw, Poland. The prepared mortars were then placed into polystyrene moulds of 4 × 4 × 16 cm, as specified in the same standard PN-EN 196-1. The synthesis of cement–fly ash composites was carried out in three stages to evaluate the effect of fly ash admixtures and the reduction of the Portland cement (42.5R) content on the compressive strength of the specimens. Table 3 illustrates the weight composition of the raw materials used to synthesise cement–fly ash composites.

The literature confirms that incorporating combustion by-products, such as fly ash, at levels of up to 50% (high-volume fly ash–HVFA) can positively affect the physicochemical properties of cement–fly ash composites. Moreover, studies indicate improvements in mechanical performance, specific surface area, and the immobilisation of heavy metal ions. Consequently, two replacement levels were selected: 25% (practical), commonly used in concrete technology, and 50% (HVFA), representing a high level of cement reduction [52,53,54].

**Figure 2:**
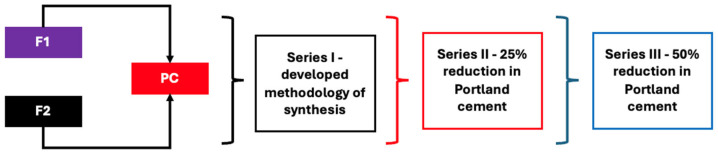
Block description of the procedure for the synthesis of cement–fly ash composites (black—Series I, red—Series II, and blue—Series III).

Figure 2 provides a schematic illustration of the synthesis procedure. Fly ash and Portland cement samples were weighed using a precision balance and homogenised for approximately 10 min to ensure uniform distribution of the components. Water was then gradually added to the dry mixture while continuously mixing until a plastic consistency was achieved. To prevent water evaporation and maintain consistent hydration during the curing process, the moulds were covered with a cling film.

The samples were left to cure at room temperature (293 ± 5 K). After 24 h, the cement mortars exhibited significant curing, evidenced by a firm consistency and the appearance of water droplets on the inner surface of the cling film. This procedure allowed systematic evaluation of the mechanical properties of composites, particularly their compressive strength, under controlled experimental conditions.

#### 2.1.2. Determination of Compressive Strength

Compressive strength measurements were performed using an automatic press designed to test the strength of cement beams, in accordance with the PN-EN 196-1 standard (MULTISERW-Morek, Marcyporęba, Poland). The composite beam was positioned on the iron plate according to the accepted standards. The test was initiated through the control panel, and the apparatus applied pressure at a constant rate of 0.031 MPa/s to the sample surface until failure occurred. The duration of the measurements varied significantly depending on the sample and synthesis method, ranging from 2 to 30 min. All compressive strength measurements were performed in triplicate (n = 3) for each sample composition and curing age.

#### 2.1.3. XRD Analysis

XRD analysis was performed using a PANalytical Empyrean diffractometer (Malvern Panalytical, Malvern, UK). The instrument used CuKα radiation at a wavelength of 1.5406 Å as the X-ray source. Data acquisition was carried out over a 2θ range of 5° to 80° with a step size of 0.013°. The measurements were carried out under ambient conditions to ensure accurate characterisation of the crystalline phases present in the samples. The configuration of the diffractometer included a high-resolution goniometer, which allowed accurate angular positioning, and a PIXcel3D detector was used to increase signal sensitivity and noise reduction. The experimental parameters were optimised to achieve a high signal-to-noise ratio, allowing detailed phase identification. The resulting diffractograms were analysed using dedicated software and reference databases to determine the phase composition, crystallite size, and lattice parameters. Phase identification from XRD diffractograms was carried out using the EVA software package release 2019 (Bruker AXS, Billerica, MA, USA) with reference to the ICDD PDF-4+ 2022 database.

#### 2.1.4. Fourier Transform Infrared Spectroscopy

Fourier transform infrared (FTIR) spectroscopy was used to identify the chemical bonds present and formed during the chemical activation of the fly ash samples in the subsequent phase of the research. This analysis was carried out using a Nicolet 6700 spectrometer (Thermo Scientific, Madison, WI, USA) equipped with an Attenuated Total Reflection (ATR) module, which allows direct measurement of the sample surface without extensive preparation.

ATR-FTIR measurements were performed over a wavenumber range of 4000–600 cm^−1^, ensuring comprehensive coverage of the vibrational modes associated with the major functional groups in the samples. The spectrometer operated at a resolution of 4 cm^−1^, and each spectrum was an average of 32 scans to improve signal clarity and reduce noise.

FTIR spectra were baseline-corrected using the OMNIC software suite version 8.2 (Thermo Scientific, Waltham, MA, USA).

#### 2.1.5. Characterisation of Porous Texture

The porous texture of the samples was characterised by measuring nitrogen adsorption/desorption isotherms at 77 K using a 3Flex sorption analyser (Micromeritics, Norcross, GA, USA). Before analysis, all samples were preconditioned, including drying at 348 K for 48 h to remove residual moisture and unreacted water to ensure accurate and reproducible results. The specific surface area was calculated using the Brunauer–Emmett–Teller (BET) method, widely recognised for its application in surface area analysis. Furthermore, the Barrett–Joyner–Halenda (BJH) method was employed to determine the pore size distribution of mesopores, and the Dubinin–Radushkevich (DR) method was applied to determine micropore volume and surface area.

Specific surface area (BET), pore volume, and pore size distribution (BJH, DR) were calculated using the MicroActive software V7.00 (Micromeritics) supplied with the 3Flex analyser.

#### 2.1.6. Thermogravimetric Analysis—TGA

Thermogravimetric analysis of the samples obtained was carried out on a TGA Q5000 V3.17 apparatus. The measuring system parameters were the same for all the composite samples analysed. Measurements were carried out in the temperature range of 25 to 900 °C with a constant argon flow of 60 mL/min and helium flow of 10 mL/min. The system temperature rise was constant at 10 K/min. Thermal analysis involves determining the change in the mass of the sample under a specific temperature regime. The results of the analysis make it possible to determine physical phenomena such as phase transitions, adsorption and desorption, and chemical reactions (oxidation and reduction).

Thermogravimetric data were processed using TA Universal Analysis software v5.5.24.

## 3. Results and Discussion

### 3.1. Measurement of Compressive Strength

**Figure 3:**
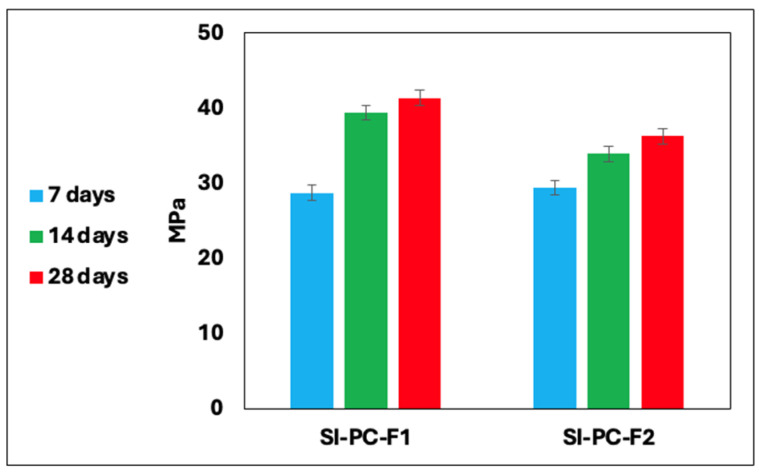
Compressive mechanical strength of cement beams series I.

**Figure 4:**
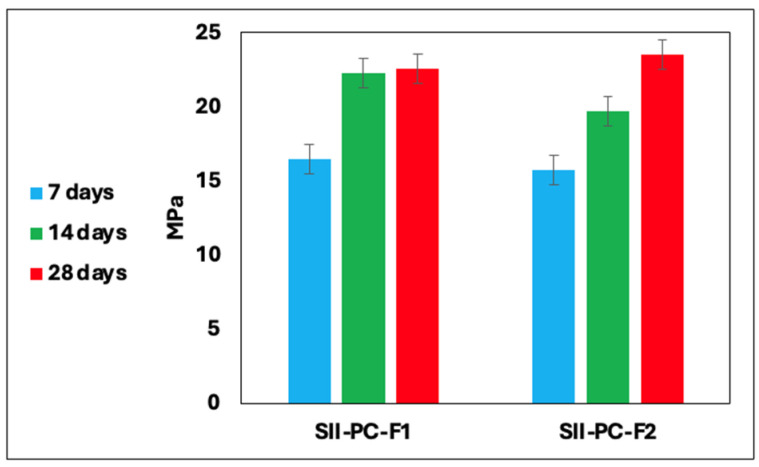
Compressive mechanical strength of cement beams series II.

**Figure 5:**
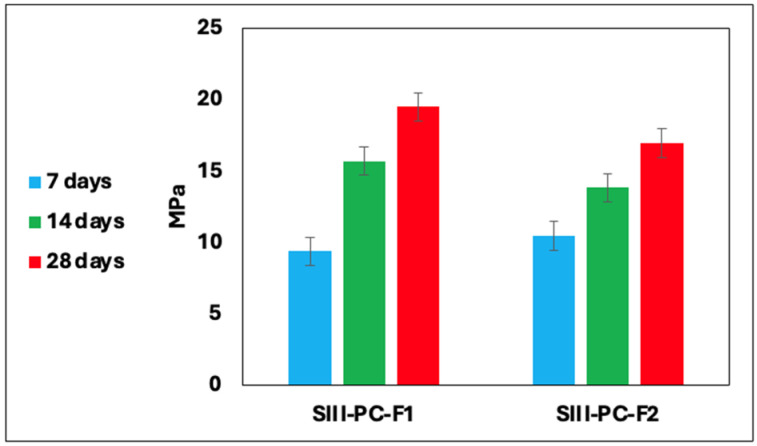
Compressive mechanical strength of cement beams series III.

The results of the mechanical strength measurements of the cement–fly ash composites are presented in Figure 3, Figure 4 and Figure 5. After seven days of curing, the samples achieved stabilisation. The highest compressive strength, reaching approximately 41 MPa, was observed in samples from Series I, particularly those containing fly ash derived from coal combustion. A reduction in cement content led to a decrease in mechanical strength. Furthermore, substituting cement with combustion by-products from various industrial sources positively influenced the physical characteristics of the final materials. Table 4 summarises the compressive strength changes of the cement–fly ash composites after 7, 14, and 28 days, presented as the mean of three independent measurements and standard deviation.

This observation agrees with the results described in the literature. Deng et al. [55] developed a method for the synthesis of a cement ash slurry using 42.5 grade cement with fly ash from incineration from an energy source added in ratios ranging from 1:0.5 to 1:3. Their study showed that the reduction of the cement content reduced the compressive strength, with the most rapid stabilisation occurring within the first 14 days. Similarly, Zhao et al. [56] investigated the replacement of 50% cement with fly ash, supplemented with nanosilica (Nano-SiO_2_, NS) and steel fibres (SFs). The inclusion of steel fibres, even at a concentration as low as 2% vol.%, significantly improved compressive strength, achieving increases exceeding 100%. In contrast, nanosilica contributed modestly to strength enhancement. The combined effect of NS and SF resulted in a strength improvement of 50–70% compared to control samples. Islam et al. [57] examined the use of fly ash as a partial substitute for cement (up to 25%), combined with crushed bricks. Their results indicate the fastest setting time within the first seven days, followed by a stabilised compressive strength. A 10% fly ash admixture was identified as the most effective proportion to achieve optimal strength. In another study, Somna et al. [58] analysed geopolymer synthesis using fly ash activated with sodium hydroxide (NaOH) at concentrations ranging from 4.5 to 16.5 mol/dm^3^. They reported a compressive strength of approximately 25 MPa at an optimal NaOH concentration of 14 mol/dm^3^. In a recent publication, Chen et al. [59] presented a novel approach to synthesise geopolymers using fly ash by chemical activation with an alkaline solution. The activation mixture comprised sodium hydroxide in a concentration range of 3 to 9 mol/dm^3^, along with Na_2_SiO_3_ in varying proportions, producing a variety of geopolymer samples with a strength ranging from 15 to 30 MPa. The methods of utilising combustion by-products as substrates in the geopolymerisation process are in accordance with the concept of a closed-loop economy, facilitating the production of materials suitable for use in the construction industry. However, they are primarily characterised by increased complexity and expense.

The approach employed in this study, using Portland cement 42.5R as the sole activating agent, stands in deliberate contrast to the conventional geopolymerisation route, which relies on concentrated solutions of sodium hydroxide or sodium silicate to dissolve the aluminosilicate network of fly ash. Research on fly ash-based geopolymers has shown that chemical and mechanical activation can significantly impact compressive strength. Shamsah et al. [60] demonstrated that geopolymers containing 5% slag, when cured at ambient temperature, can achieve a compressive strength of up to 61 MPa, thanks to the synergistic N-A-S-H gel reaction. Xu et al. [61] in their research, developed a method for the thermal alkaline activation of combustion by-products. The study showed that the system’s high temperature promotes the dissolution of the glassy phase in fly ash, resulting in the formation of a cementitious matrix. The compressive strengths achieved in Series I of this study (up to 41 MPa at 28 days) are comparable to those reported for NaOH-activated geopolymers at their optimal concentration, while eliminating the health and environmental risks associated with concentrated alkaline solutions. This confirms the viability of Portland cement as a safer and operationally simpler alternative activator for fly ash in industrial applications, fully aligned with circular economy principles.

### 3.2. X-Ray Diffraction (XRD) Analysis

**Figure 6:**
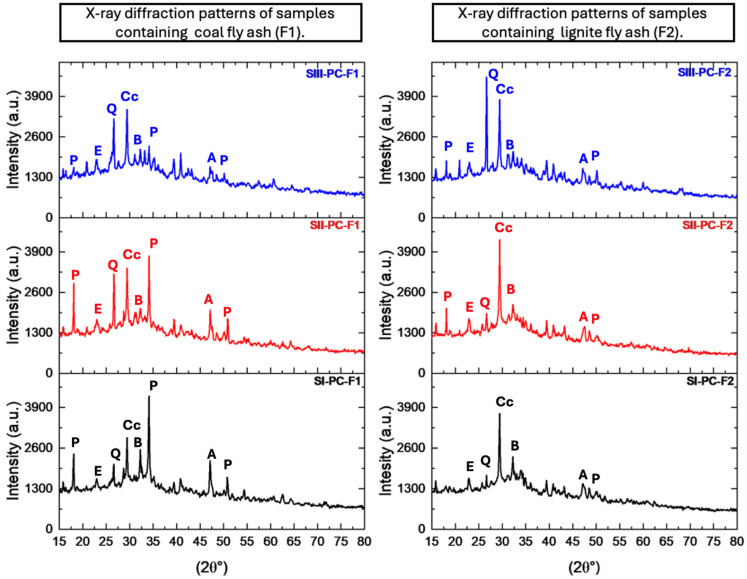
X-ray diffraction patterns of samples containing coal fly ash (F1) and lignite fly ash (F2) in a Portland cement (PC) matrix (black—Series I, red—Series II, and blue—Series III): Cc—calcite; P—portlandite; A—alite; B—belite; E—ettringite; Q—quartz.

The objective of the XRD analysis was to identify the crystalline phases present in the samples after chemical activation (Figure 6). The analysis was carried out in two stages, with each stage examining specific material compositions:

- In the first stage, a mixture of coal-derived fly ash (F1) and cement (PC) was examined;
- In the second stage, a mixture of lignite-derived fly ash (F2) and cement (PC) was analysed.

Across both stages, the diffractograms revealed recurring crystalline phases, primarily composed of silicon, aluminium, and calcium compounds. The presence and intensity of the reflections corresponding to calcite, portlandite, alite, belite, ettringite, and quartz varied depending on the composition and ratio of the components in each sample series. Similar observations have been reported in other studies, where fly ash was used as a pozzolanic additive in cement mortars. X-ray diffraction (XRD) analyses in these works revealed the presence of key crystalline phases such as alite (C_3_S), belite (C_2_S), portlandite (Ca(OH)_2_), and ettringite (AFt), indicating that the addition of fly ash influences the hydration products and the development of the microstructure [62,63]. These crystalline compounds are crucial for determining the mechanical and chemical properties of cement–fly ash composites. Changes in cement content and type of supplementary materials (e.g., coal fly ash and lignite fly ash) influenced the formation and intensity of these phases, reflecting their contribution to the overall structural integrity and durability of composites.

The primary minerals and compounds forming the crystal lattice of cement–fly ash composites include calcite, portlandite, and belite. As belite (Ca_2_SiO_4_, noted C_2_S in the cement nomenclature) is a key mineral in traditional Portland cement, its content was the highest in all test series. The reflections of belite, particularly at an angle of 32°, were the most intense in the first series of measurements, but their intensity decreased as the cement content decreased.

Portlandite-related reflections were detected at 17°, 34° and 52°; however, in the case of the composite where fly ash was used from hard coal combustion, its intensity was much higher than in the composite with fly ash from the thermal processing of lignite. A consistent presence of ettringite (Ca_6_Al_2_(SO_4_)_3_(OH)_12_·26H_2_O) was observed in all samples analysed. In samples containing combustion of fly ash coal, the intensity of ettringite reflections at 23° increased with decreasing Portland cement content. In contrast, the intensity of the crystalline phase remained relatively stable in samples containing lignite fly ash (F2). Ettringite is formed by the chemical reaction between tricalcium aluminate (C_3_A) and gypsum (CaSO_4_·2H_2_O), both of which are present in significant amounts in fly ash. The synthesis of cement–fly ash composites was carried out in an aqueous environment, which facilitated the formation of this crystalline phase.

### 3.3. FTIR Analysis

**Figure 7:**
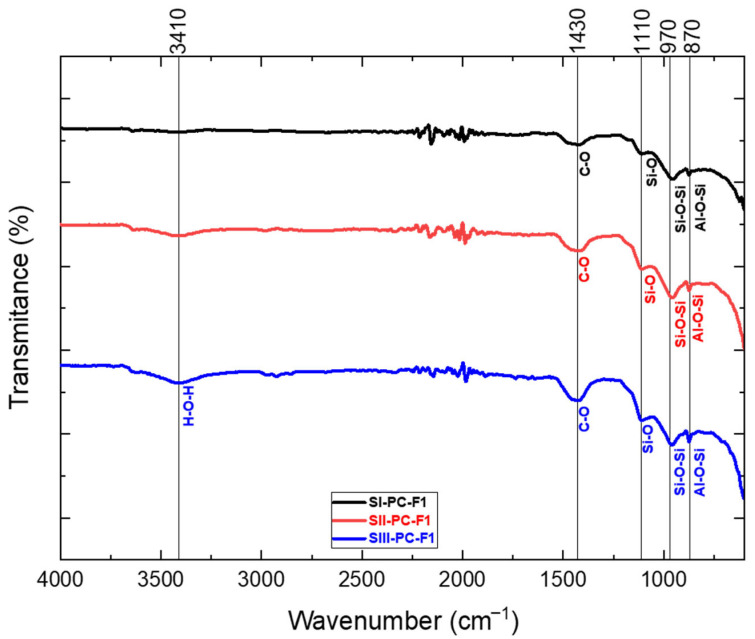
FTIR analysis for samples containing fly ash from hard coal.

**Figure 8:**
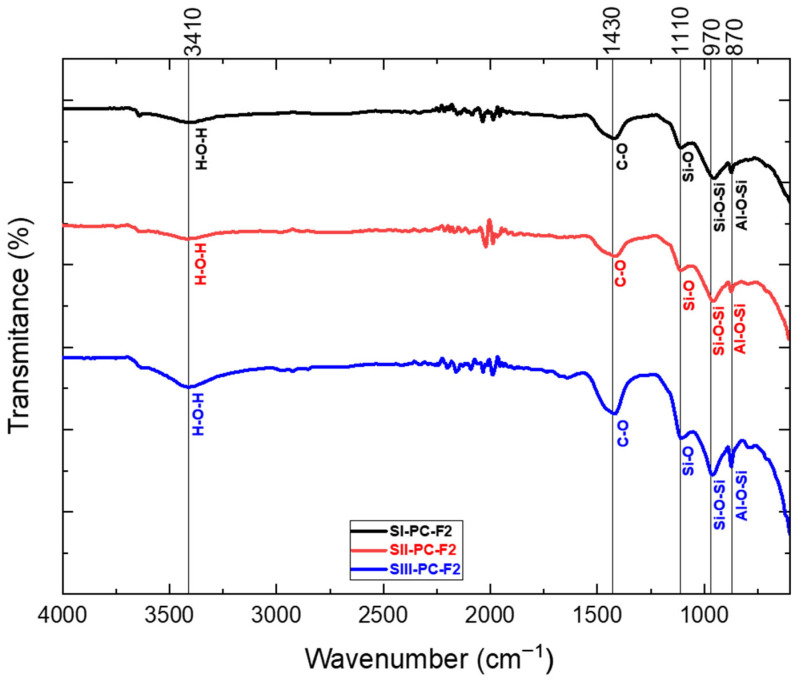
FTIR analysis for samples containing fly ash from lignite.

The purpose of the analysis was to identify the characteristic spectra of the chemical bonds formed during the chemical reaction of fly ash from both the power sector and Portland cement. The resulting spectra, presented in Figure 7 and Figure 8, provide information on the bonding structures within the cement–fly ash composite samples. Each spectrum is defined not only by its shape but also by the intensity of the absorption bands at specific wavenumbers (cm^−1^), reflecting the presence of different functional groups and chemical interactions.

- Al-O-Si (875 cm^−1^)—rations coming from the AlO_4_*^−^* group, confirming the initiation of a chemical reaction, the dissolution of the aluminosilicates contained in the sample [64].
- Si-O-Si (970 cm^−1^)—stretching vibration [65,66].
- Si-O (1110 cm^−1^)—the chemical reaction between Portland cement and fly ash demonstrating pozzolanic properties increases the content of the C-S-H phase, thus reducing Ca(OH)_2_ [67].
- C-O (1410 cm^−1^)—asymmetric stretching, peaks originating from carbonate ions (CO_3_^2−^) and more specifically from calcite [68].
- H-O-H (3450 cm^−1^)—stretching vibration [69].

The application of infrared (IR) spectroscopy allowed the precise identification and localisation of the chemical bonds present in the cement–fly ash composite samples. Despite the substantial amount of water used during the synthesis process, the resulting IR spectra did not show prominent stretching bands within the 3400–3500 cm^−1^ range. This observation suggests that the curing period was sufficiently prolonged, allowing the water within the mortar matrix to become adsorbed either by the formed chemical bonds or by their residual structures, exceeding the time frame of primary chemical reactions.

A defining characteristic of construction materials, including cement and cement-based concrete, is the considerable presence of chemical compounds containing silicon and aluminium. This is supported by the observed spectra corresponding to Si-O-Si and Al-O-Si bonds in the analysed samples. The detected CO bonds are likely attributable to calcium and magnesium carbonates.

As the content of cement 42.5R (PC) decreased, the IR spectra revealed progressively extended absorption bands accompanied by a corresponding decrease in transmittance values.

### 3.4. Porous Texture Based on Nitrogen Adsorption Isotherms at 77 K

The analysis of the porous texture of the cement–fly ash composites was carried out using nitrogen adsorption isotherms at 77 K. This method provides detailed insight into the surface properties and pore structure of materials, which are critical for understanding their sorption capacity and overall performance in various applications. The introduction of fly ash (F1, F2) into Portland cement (PC) resulted in composite materials with modified textural properties, which influenced their adsorption and structural characteristics.

The porous texture analysis involved the determination of key textural parameters from the nitrogen adsorption isotherms:

- S_BET_ (m^2^/g)—Specific surface area determined using the Brunauer–Emmett–Teller (BET) method, which characterizes the total surface area available for adsorption.
- S_DR_ (m^2^/g)—Surface area calculated using the Dubinin–Radushkevich (DR) method, primarily associated with microporous structures.
- S_BJH_ (m^2^/g)—Surface area derived from the Barrett–Joyner–Halenda (BJH) method, focused on mesopores.
- V_DR_ (cm^3^/g)—Pore volume calculated using the Dubinin–Radushkevich (DR) method, associated with microporosity.
- V_BJH_ (cm^3^/g)—Pore volume determined by the BJH method, indicating the mesoporous fraction of the material.
- V_total_ (cm^3^/g)—Total pore volume, representing the cumulative contribution of all pore sizes present in the material.

Reducing the cement content and replacing it with fly ash (F1 and F2) generally resulted in an increase in pore area and volume. In each of the three test series, samples containing fly ash from lignite combustion (F2) had a larger specific surface area BET (SBET) than those using fly ash from bituminous coal combustion (F1) as substrate. In series III, the sample (SIII-PC-F2) showed the highest total pore volume (Vtotal) and mesopore surface area (SBJH), confirming the beneficial effect of the addition of lignite fly ash with a high calcium oxide content. Furthermore, the micropore surface area (SDR) reached maximum values in the samples with a significant reduction in the cement content—50%. Taken together, these results show that the replacement of cement with fly ash optimises both microporous and mesoporous structures, increasing the adsorption capacity and improving the textural properties of the composites. The results of the textural parameters for the obtained cement–fly ash composites are summarized in Table 5.

**Figure 9:**
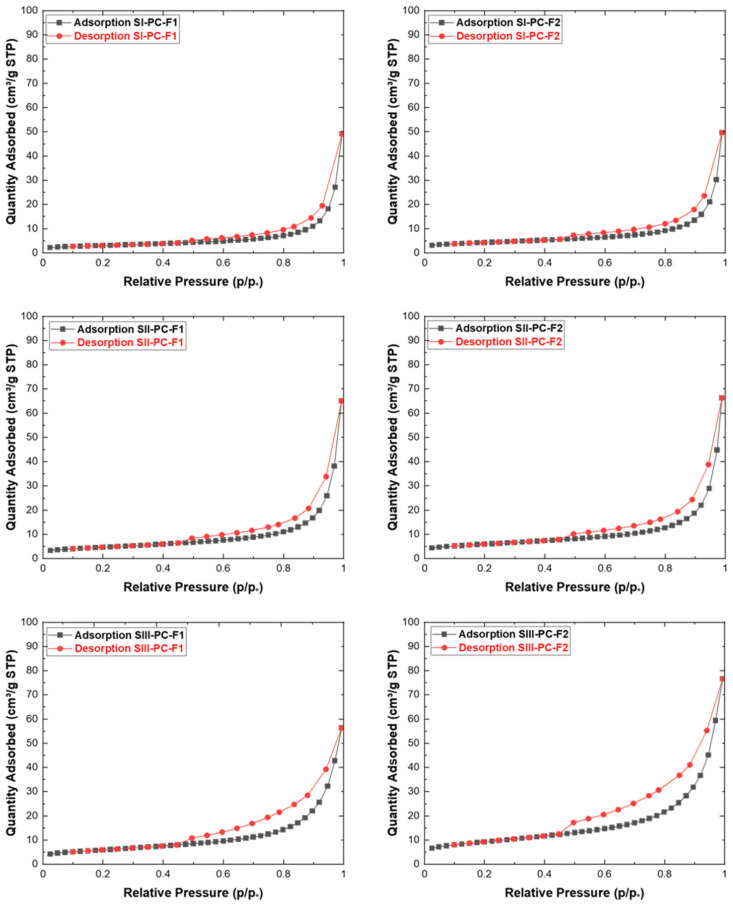
Nitrogen adsorption–desorption isotherms at 77 K for the cement–fly ash composite samples.

Figure 9 presents the nitrogen adsorption–desorption isotherms at 77 K for the cement–fly ash composite samples. According to the IUPAC classification, all observed isotherms correspond to type IV, which is characteristic of mesoporous materials. This type of isotherm indicates the presence of well-developed mesopores, where capillary condensation occurs at higher relative pressures. The hysteresis loops observed in the desorption branch further confirm the mesoporous nature of the composites. The shape and slope of the adsorption branches suggest differences in pore size distribution and pore geometry in the set of the analysed samples.

**Figure 10:**
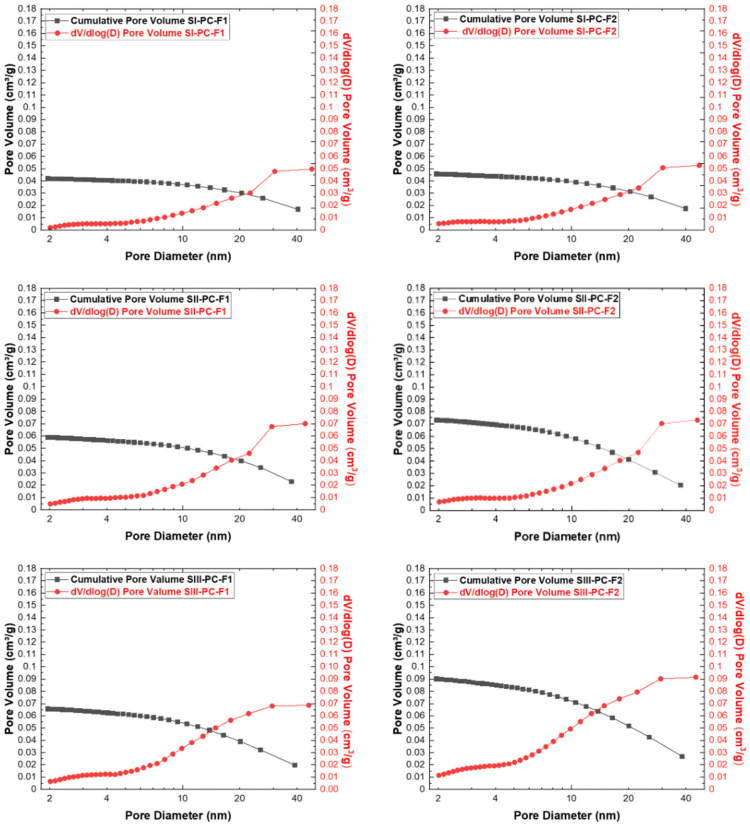
BJH analysis for the cement–fly ash composites obtained.

The pore size distribution (PSD) of the mesopores in the cement–fly ash composite samples was determined using the BJH method (Barrett–Joyner–Halenda) and is shown in Figure 10. This method, based on the Kelvin equation, allows the characterisation of the mesopore size and volume distribution by analysing the adsorption and desorption branches of the nitrogen isotherms. The results show distinct differences in the distribution patterns of pore sizes depending on the type of additive and the degree of cement substitution. PSD curves indicate the presence of a well-developed mesoporous network, with variations in pore diameter and volume reflecting the influence of the origin and structure of the additive. Samples containing fly ash from lignite combustion have a wider range of mesopores. This analysis highlights the important role of cement-replacement additives in the design of the mesoporous structure of composites, which directly affects their adsorption efficiency and sorption capacity.

### 3.5. Thermogravimetric Analysis

Thermogravimetric analysis (TGA) is a technique that can be used to determine the physical and chemical properties of cement–fly ash composites. This analysis allows for the tracking of changes in the weight of the sample as a function of temperature, which is related to various physicochemical processes such as hydration, dehydration, or chemical reactions that occur during heating. In the specific case of cement–fly ash composites, thermogravimetric curves can be characterised by several characteristic points related to the crystalline phases that result from the synthesis of these materials. The following are some of the most common points and processes:

- The first is the deflection of the curve at temperatures above 373 K. This is attributed to the desorption of water molecules that remain after the hydration process of the cement pastes.
- Subsequently, the curves exhibit a bend within the temperature range of 393 to 673 K, corresponding to the dehydration process of the C-S-H phase and ettringite, which was also identified in the cement–fly ash composites obtained.
- Within the temperature range of 673 to 873 K, thermal decomposition of portlandite occurs.
- The final stage is the decomposition of calcium carbonate, which occurs above 923 K, resulting in the formation of calcium oxide and carbon dioxide [70].

**Figure 11:**
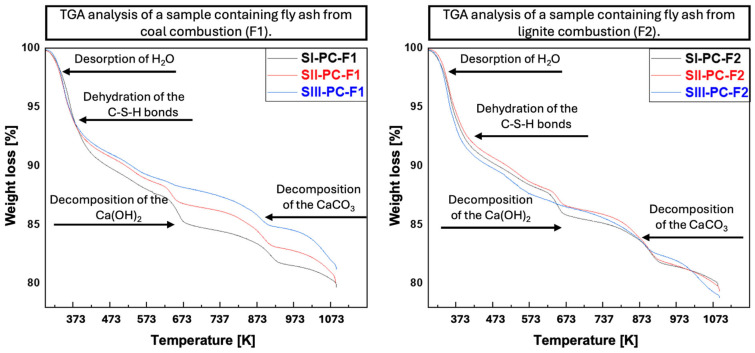
TGA of cement–fly ash composites.

The thermogravimetric curves of the cement–fly ash composites obtained are shown in Figure 11. It is evident that, despite employing identical parameters for the TGA measurements, the curves obtained exhibit differences. In the case of the sample containing fly ash from coal combustion (F1), the sample showed the highest stabilisation in an elevated temperature environment after reduction with 50% Portland cement (SIII-PC-F1), while in the sample containing fly ash F2 (from lignite combustion), the sample showed the highest resistance to temperature influence by the sample from the second series of measurements—SII-PC-F2. The results of TGA of the F1 fly ash series indicated that the greatest mass loss in the calcium hydroxide decomposition region occurred in the sample with the highest ash content. The incorporation of fly ash was found to enhance compressive strength, attributable to the introduction of silicon and aluminium oxides, as well as minor amounts of calcium oxide capable of hydration to calcium hydroxide. It can be assumed that the calcium compounds present in the cement are sufficient to react with the oxides from the ash, promoting the formation of aluminosilicates. Samples containing fly ash from lignite (F2) exhibited significantly higher calcium oxide content compared to those with fly ash from hard coal (F1). As can be seen in Figure 10, this reduces the efficiency of the hydration process. The mass loss in the temperature range of 673–873 K is almost identical for the SI-PC-F2, SII-PC-F2 and SIII-PC-F2 samples. Sun et al. [71] developed a method for using fly ash as a 30% additive in cement mortar with a water-to-binder ratio of 2:5. The authors observed that over a 90-day curing period, samples containing fly ash consistently showed lower mass loss than those without the additive, indicating a slower and more limited development of hydration phases due to the pozzolanic effect of the fly ash.

## 4. Summary and Conclusions

In this study, a method for the utilisation of fly ash as a raw material in the synthesis of cement–fly ash composites was developed. The conventional approach based on concentrated alkaline activators (NaOH, KOH) was replaced with Portland cement (42.5R) as a safer and simpler activating agent. The proposed approach enables a reduction in cement consumption while maintaining mechanical performance and controlling the physicochemical properties of the resulting materials. The main findings are as follows:

- Fly ash enables partial replacement of Portland cement up to 50%, while maintaining compressive strength up to 41 MPa, confirming its applicability in cementitious systems.
- The chemical composition of the fly ash (F1 and F2), rich in SiO_2_, Al_2_O_3_, and CaO, supports pozzolanic reactivity and contributes to the formation of stable cementitious phases.
- XRD analysis showed that fly ash content influences phase composition, with variations in the intensity of crystalline phases such as portlandite, ettringite, and belite.
- FTIR results confirmed the formation of Si-O-Si, Al-O-Si, and C-S-H structures, indicating ongoing polymerisation and development of the cementitious matrix.
- TGA confirmed the progress of pozzolanic reactions, reflected by changes in Ca(OH)_2_ decomposition and thermal stability of the composites.
- Reducing cement content increased specific surface area BET (S_BET_) and pore volume, with lignite-derived fly ash (F2) showing the highest mesoporosity and surface area S_BET_. The porous structure and phase composition are strongly dependent on fly ash type and content, affecting the overall physicochemical properties of the composites.

The use of fly ash enables partial replacement of natural raw materials and supports the utilisation of industrial by-products. The environmental relevance of this approach is supported by the literature data, indicating that fly ash incorporation may contribute to reducing the carbon footprint of cement-based materials, primarily through reduced clinker content. From an economic perspective, it may also lower material costs and waste management requirements. The proposed method provides a simple alternative to conventional alkaline activation routes. Further research could address long-term durability, e.g., frost and sulphate resistance, leaching behaviour, and environmental assessment of the developed composites.

## Figures and Tables

**Table 1:** Economic aspects of fly ash from hard coal and lignite.

Fly Ash	Main Economic Uses	Economic Advantages	Economic Limitations
F	Concrete, cement, geopolymers, zeolites	Higher market value, reduced clinker costs	Declining availability in countries moving away from hard coal
C	Soil remediation, recovery of rare earth metals	Low price, high local availability	Lower market value, limited use in cement production

**Table 2:** Physicochemical properties of cement according to standards: EN 196-1, EN 196-2, EN 196-3 and EN 196-6 [48,49,50,51].

Property	Value	Test Method
Compressive strength, 2 days (MPa)	≥20	EN 196-1
Compressive strength, 28 days (MPa)	42.5–62.5	EN 196-1
Initial setting time (min)	≥60	EN 196-3
Soundness–expansion (mm)	≤10	EN 196-3
Specific surface area—Blaine (m^2^/kg)	350–450	EN 196-6
SiO_2_ (wt%)	19–21	EN 196-2
Al_2_O_3_ (wt%)	4–6	EN 196-2
Fe_2_O_3_ (wt%)	2–4	EN 196-2
CaO (wt%)	62–65	EN 196-2
MgO (wt%)	<5	EN 196-2
SO_3_ (wt%)	<4	EN 196-2
Na_2_O equivalent (wt%)	<0.6	EN 196-2
Loss on ignition (wt%)	<5	EN 196-2

**Table 3:** Summary of the weights of the raw materials used in the synthesis of cement–ash composites.

Sample	PC [g]	F1 [g]	F2 [g]	Total [g]
SI-PC-F1	250.00	150.00	0.00	400.00
SI-PC-F2	250.00	0.00	150.00	400.00
SII-PC-F1	187.50	212.50	0.00	400.00
SII-PC-F2	187.50	0.00	212.50	400.00
SIII-PC-F1	125.00	275.00	0.00	400.00
SIII-PC-F2	125.00	0.00	275.00	400.00

**Table 4:** Summary of compressive strength measurements.

Sample	Series	7 days	*σ*_7days_	14 days	*σ*_14days_	28 days	*σ*_28days_
SI-PC-F1	I	28.72	1.06	39.42	1.01	41.36	1.05
SI-PC-F2	I	29.42	1.01	33.91	1.09	36.27	1.10
SII-PC-F1	II	16.47	0.97	22.29	0.94	22.60	1.01
SII-PC-F2	II	15.72	0.92	19.73	0.99	23.51	0.99
SIII-PC-F1	III	9.37	0.98	15.71	0.99	19.49	0.99
SIII-PC-F2	III	10.48	0.76	13.83	0.83	16.97	0.91

**Table 5:** Results of the textural parameters for the cement–fly ash composites.

Sample	S_BET_ (m^2^/g)	V_DR_ (cm^3^/g)	V_BJH_ (cm^3^/g)	S_DR_ (m^2^/g)	S_BJH_ (m^2^/g)	V_total_ (cm^3^/g)
SI-PC-F1	10.77	0.004	0.041	12.81	9.20	0.076
SI-PC-F2	15.12	0.006	0.047	17.89	11.26	0.077
SII-PC-F1	16.44	0.007	0.059	19.33	14.54	0.086
SII-PC-F2	21.03	0.009	0.068	24.87	15.99	0.103
SIII-PC-F1	20.94	0.009	0.065	24.56	18.32	0.087
SIII-PC-F2	32.77	0.014	0.090	38.65	26.95	0.119

## Data Availability

The original contributions presented in this study are included in the article. Further inquiries can be directed to the corresponding author.
